# Dark-light cycle disrupts bone metabolism and suppresses joint deterioration in osteoarthritic rats

**DOI:** 10.1186/s13075-022-02832-8

**Published:** 2022-06-28

**Authors:** Xiaopeng Song, Mingchao Zhao, Jilang Tang, Tianwen Ma, Hui Bai, Xinyu Wang, Lin Liu, Ting Li, Xinyu Xu, Xuanbo Sheng, Binger Zhao, Yingying Wang, Tiantian Wang, Yingchao Guo, Xinmin Zhang, Li Gao

**Affiliations:** Heilongjiang Key Laboratory of Animal Disease Pathogenesis and Comparative Medicine, College of Veterinary Medicine, Northeast Agriculture University, 600, Changjiang Road, Harbin, 150030 China

**Keywords:** Light, Circadian, Osteoarthritis, Rat, Joint, Bone, Metabolism

## Abstract

**Background:**

Light alteration affects the internal environment and metabolic homeostasis of the body through circadian rhythm disorders (CRD). CRD is one of the factors that induce and accelerate osteoarthritis (OA). Therefore, the aim of this study was to evaluate the effects of continuous dark-light (DL) cycle on joint inflammation, bone structure, and metabolism in normal and OA Sprague-Dawley (SD) rats.

**Methods:**

Interleukin (IL)-1β, IL-6, inducible nitric oxide synthase (iNOS), and tumor necrosis factor (TNF)-α were used to evaluate the systemic inflammation in rats. The pathological changes and inflammatory reactions of the cartilage and synovium of the knee joint in rats were evaluated by Safranin O-fast green and immunological staining. Bone turnover was assessed by histomorphometry and μCT scanning, as well as bone metabolism markers and proteins. The expression changes of clock proteins BMAL1, NR1D1, PER3, and CRY1 in representative tissues were detected by western blotting.

**Results:**

DL cycle significantly inhibited body weight gain in normal and OA rats. The levels of proinflammatory factors in the peripheral blood circulation and degradation enzymes in the cartilage were significantly decreased in OA+DL rats. DL cycle significantly destroyed the structure of subchondral bone in hindlimbs of OA rats and reduced trabecular bone numbers. The decrease of bone mineral density (BMD), percent bone volume with respect to total bone volume (BV/TV), trabecular number (TB.N), osteoclast number, and mineralization could also be found. The ratio of the receptor activator of nuclear factor-kappa B ligand/osteoprotegerin (RANKL/OPG) in the bone marrow of OA rats was markedly increased under DL, along with the activation of the mononuclear/phagocyte system. The expression of representative clock proteins and genes BMAL1, PER3, and CRY1 were markedly changed in the tissues of OA+DL rats.

**Conclusions:**

These results suggested that DL cycle dampened the arthritis and promoted bone resorption and bone mass loss.

**Graphical abstract:**

DL cycle affects bone turnover by regulating osteoclast production in osteoarthritic rats.
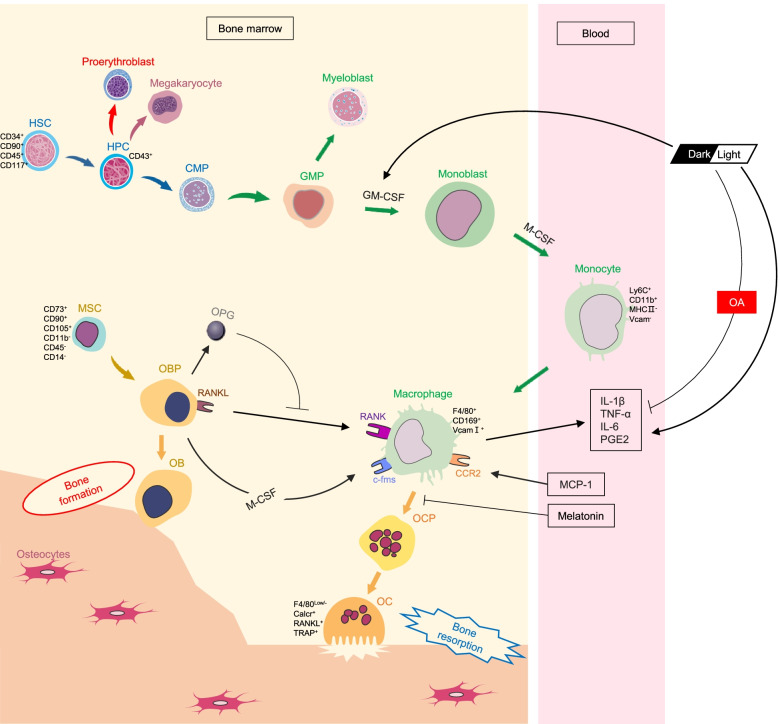

**Supplementary Information:**

The online version contains supplementary material available at 10.1186/s13075-022-02832-8.

## Introduction

The circadian clock consists of a central clock and peripheral clock, and light is one of the many cues that can entrain the circadian clock [1]. The light signal is projected to the retina and passed into the suprachiasmatic nucleus (SCN), the master circadian oscillator of mammals, through the retina hypothalamic tract (RHT). This causes a phase shift of the circadian rhythm, which synchronizes the body's internal circadian rhythm with the light and dark (LD) cycle of the external environment [2]. Changes in the pattern or intensity of light lead to a desynchronization between the sleep-wake rhythm and the circadian cycle, which can induce body metabolic disorders and diseases. Previous studies suggested that shift work increased the risk of chronic metabolic-related diseases, such as cardiovascular disease, steatohepatitis, and type 2 diabetes [3–5].

Osteoarthritis (OA) is a common form of osteoarthrosis. Typical lesions of OA include cartilage loss, increased subchondral bone thickness, tidemark replication, and decreased subchondral trabecular bone mass [6]. The circadian rhythm, along with species, age, gender, and trauma, represent important factors in the influence of arthritis [7]. Rheumatoid arthritis (RA) and OA come are considered as the “chronobiological diseases,” exhibiting diurnal or 24 h pattern in symptom intensity [8]. Circadian desynchronization increases the risk of OA in shift workers [9]. A recent study found that 24 h continuous light (LL) induces an inflammatory microenvironment in osteoarthritic joints, and even causes trabecular bone loss [10]. The environmental disruption of the circadian rhythms induces OA-like changes in mouse knee joint [11]. Clock genes regulate endochondral ossification during the bone development of mice after birth, as revealed by the body length and longitudinal length of the tibia and femur in brain and muscle Arnt-like protein 1 (*BMAL1)*^*-/-*^ mice, which are significantly shorter than those in *BMAL1*^*+/+*^ mice [12, 13]. Additionally, an aging muscle phenotype and reduced specific force in the extensor digitorum longus muscle could be found in BMAL1 KO mice [14]. These results demonstrate the close interaction between the circadian rhythm and musculoskeletal system. Therefore, the aim of the study was to determine the effects of environmental lighting conditions on joint inflammation and bone metabolism in normal and OA rats to prevent bone mass loss and improve the prognosis of OA.

## Methods

### Animals, OA induction, and groups

**Fig. 1:**
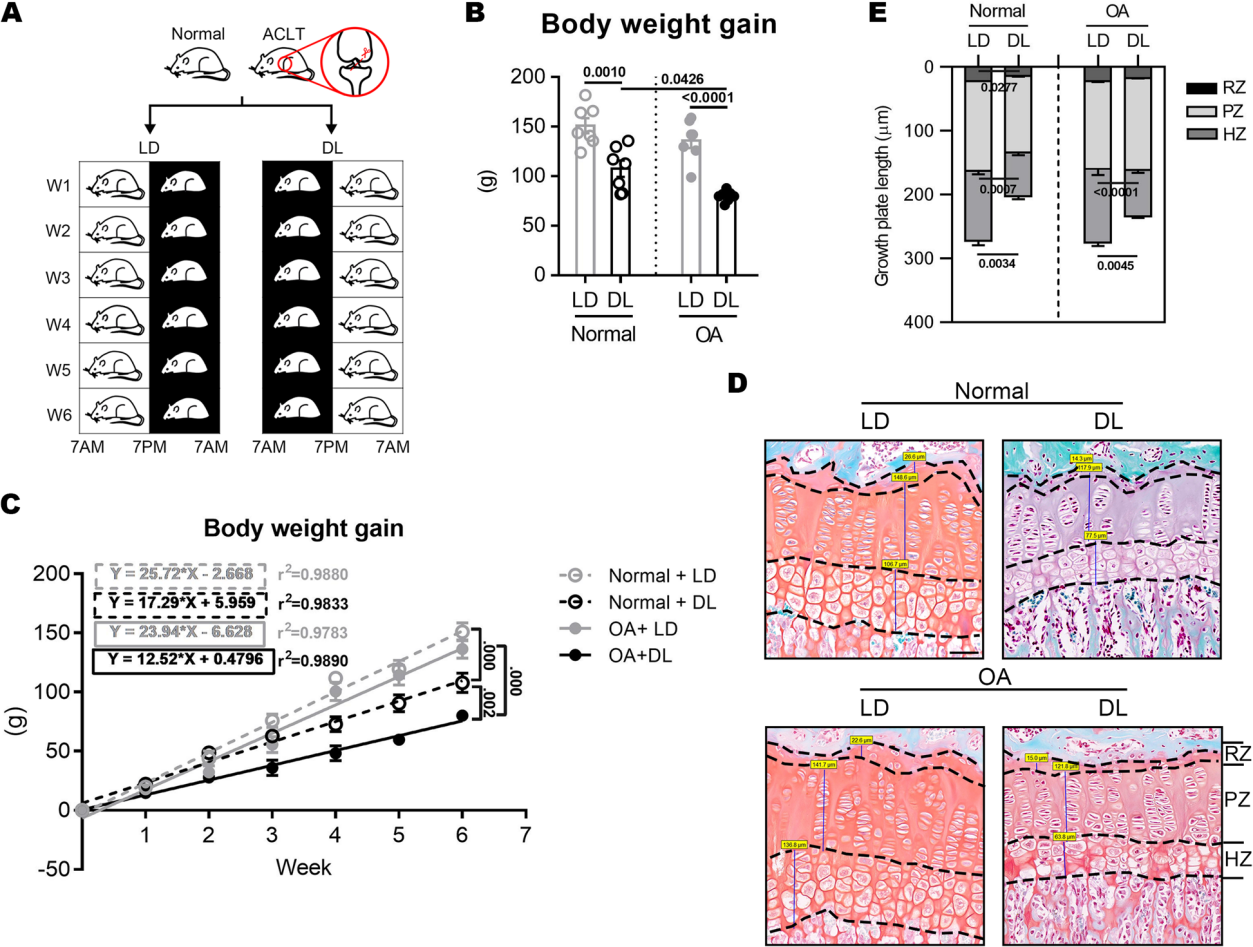
Body growth of normal and OA rats under LD and DL cycle. **A** LD and DL cycle protocol. **B** Body weight gain of rats at the end of study. Two-way ANOVA: LD factor: *F*_(1, 24)_ = 51.81, *P* < 0.0001; model factor: *F*_(1, 24)_ = 9.408, *P* = 0.0053; *n* = 7 per group. **C** Linear regression of body weight gain and weeks in each group. **D** Safranin O and fast green staining for tibia growth plates of normal and OA rats. RZ, resting zone; PZ, proliferating zone; HZ, hypertrophy zone. Scale bar, 50 μm. Two-way ANOVA: Total length: LD factor: *F*_(1, 8)_ = 25.52, *P* = 0.0010; RZ: LD factor: *F*_(1, 8)_ = 13.94, *P* = 0.0058; PZ: LD × model: *F*_(1, 8)_ = 8.507, *P* = 0.0194; HZ: LD factor: *F*_(1, 8)_ = 38.25, *P* = 0.0003; *n* = 3 per group

A total of 60 adolescent (8–10 weeks) male Sprague-Dawley (SD) rats were purchased from Liaoning Changsheng biotechnology corporation. Room temperature (24 ± 3 °C), humidity (40–70%), noise (≤ 40 dB), light intensity (300Lux), and light cycle (control) was used as the standard conditions. Four groups of rats (15 in each group, randomly selected) were used for the experiments conducted under entrained conditions [15]. Two groups of normal (sham) rats and two groups of OA rats treated with anterior cruciate ligament transection (ACLT) [16]. The ACL of right knee was broken by micro-shearing under a microsurgical microscope (Corder Optics ad electronics Co., Ltd., Chengdu, China) without damaging the articular cartilage. And for sham rats, only the joint capsule of right knee was exposed and the ACL was not severed. The entrained experiments were performed by subjecting the rats to a 12 h light/dark (L/D 12/12) cycle, with lights on at 7 A.M. and lights off at 7 P.M. with no limitation of food intake time. Dark-light (DL) cycle consisted of lights on at 7 P.M. and lights off at 7 A.M. All groups were sacrificed by decapitation at the end of the sixth week (Fig. 1A).

### Decalcification and histological staining

Six right knee joints of rats in each group were subjected to shaker decalcification at a constant temperature (25–30 °C), blocked and sliced (Leika RM2016, China). The staining with fast green and Safranin O solution was performed. Orange-red cartilage and green bone formation were observed under the microscope (NIKON ECLIPSE E100, Japan), and images were taken for analysis. Cartilage and synovial scoring criteria were observed and evaluated as previously described [17, 18]. The length of growth plate was measured by CaseViewer 2.4.0 (3DHISTECH Ltd., Hungary).

### Immunohistochemical and immunofluorescence analysis

As regards the immunohistochemical analysis, the incubation was performed overnight at 4 °C with the following primary antibodies: BMAL1 (1:300, Absin, China), matrix degradation enzymes matrix metalloproteinase-3 (MMP-3) (1:400, Servicebio, China), MMP-13 (1:500, Absin, China), a disintegrin and metalloproteinase with thrombospondin motifs 4 (ADAMTS-4) (1:500, Servicebio, China), Collagen II (1:300, Servicebio, China), and osteoprotegerin (OPG) (1:200, Servicebio, China). The tissues were treated with secondary antibodies (HRP labeled) of the corresponding species of the primary antibody and incubated. The slices were dehydrated, sealed, and examined under the microscope (NIKON ECLIPSE E100, Japan). As regards the immunofluorescence analysis, the incubation was performed overnight with the following primary antibodies, CD206 (1:500, Absin, China), Ki67 (1:500, Servicebio, China), F4/80 (1:500, Servicebio, China), receptor activator of nuclear factor-kappa B ligand (RANKL) (1:2000, Absin, China), and OPG (1:200, Servicebio, China). The tissues were treated with the secondary antibody and DAPI solution in the dark. Images were taken by fluorescent microscopy (NIKON ECLIPSE C1, Japan).

### Bone histomorphometry and μCT scanning

The slides were incubated with filtered tartrate-resistant acid phosphatase (Trap) (G1050, Servicebio, China) solution, followed by hematoxylin to stain the nucleus. The sections were scanned with a Pannoramic section scanner (3DHISTECH, Hungary). The number of osteoclasts per unit area was calculated using Image-Pro Plus 6.0 analysis software (Media Cybemetics, USA).

Bruker Micro-CT Skyscan 1276 system (Kontich, Belgium) with an isotropic voxel size of 10 μm was used for micro-CT analysis to image the trabecular bone of the subchondral bone of the femur and tibia. The scan condition included an X-ray tube potential of 85 kV, an X-ray intensity of 200 μA, and an exposure time of 384 ms. Reconstruction was performed using Nrecon (version 1.7.4.2). 3D images were obtained from contoured 2D images by methods based on the distance transformation of the grayscale of the original images (CTvox; version 3.3.0). 3D and 2D analyze were performed using the software CT Analyser (version 1.18.8.0).

### Undecalcified bone Von Kossa staining

Three rat knee joints of each group were fixed in 4% PFA and then dehydrated. Von Kossa staining solution was immediately added to the tissue after the sections were rehydrated in alcohol and continuously subjected to ultraviolet rays. After hematoxylin staining, differentiation, and blue returning, the sections were dehydrated and stained with eosin solution following by mounting.

### Enzyme-linked immunosorbent assay of the serum

On the last day of each week during the experiment, 0.5 mL whole blood was collected from the tail vein of the rats for growth hormone (GH) detection, while at the end of the study, the whole blood was collected from the hearts for other active substance detection. The serum was extracted after centrifugation at 3000 rpm/min for 10 min. ELISA was performed using the following antibodies: rat tumor necrosis factor α (TNF-α), interleukin-1β (IL-1β), IL-6, C-terminal telopeptides of collagen type II (CTX-II), cartilage oligomeric matrix protein (COMP), melatonin (MT), β-isomerized C-terminal telopeptides (β-CTx), N-terminal propeptide of type 1 collagen (P1NP), tartrate-resistant acid phosphatase 5b (TRACP-5b), bone alkaline phosphatase (BALP), monocyte chemoattractant protein-1 (MCP-1), granulocyte-macrophage colony stimulating factor (GM-CSF), and M-CSF. These ELISA kits were all purchased from MEIMIAN (China), and inducible nitric oxide synthase (iNOS) and GH were purchased from NJJCBIO (China). The absorbance of each antibody was measured at 450 nm in a microplate reader. ELISAcalc software was used to calculate the regression equation of the standard linear curve according to the concentration and OD value, and a four-parameter logistic curve was selected as fitting model.

### Sodium dodecyl sulfate-polyacrylamide gel electrophoresis and western blotting

Total protein samples from tissues (heart, liver, spleen, lung, and kidney) of OA rats were extracted, then equal amounts (25 μg) of proteins were separated onto SDS-PAGE and transferred to nitrocellulose (NC) filter membrane. The membrane was treated with the following primary antibodies: BMAL1 (1:1000, Absin, China), nuclear receptor subfamily 1 group D member 1 (NR1D1) (1:500, Proteintech, China), cryptochrome 1 (CRY1) (1:500, Proteintech, China), Period 3 (PER3) (1:500, Proteintech, China), and glyceraldehyde-3-phosphate dehydrogenase (GAPDH) (1:750, Zsbio, China). Next, the membrane was treated with goat/mouse anti-rabbit immunoglobulin G (IgG) secondary antibody coupled with horseradish peroxidase. The blots were developed using the ECL system (Tanon-5200; China), and the intensity of the gray bands was quantified using Image J software (1.53c; USA).

### Real-time PCR

Total RNA of tissues (heart, liver, spleen, lung, and kidney) of OA rats was uniformly extracted using RNA simple total RNA kit (TIANGEN, China) and reverse transcribed into cDNA (1 μg/mL) using GoScript^TM^ reverse transcription mix (Promega, USA). The mix of Platinum® SYBR Green qPCR supermix (Invitrogen, USA), primers, and cDNA was subjected to 40 amplification cycles. Primer sequences are listed in Table 1. The intensity of the fluorescence signal during the entire PCR process was monitored using LightCycler®480 (Roche, Germany) to obtain the cycle threshold (Ct).

**Table 1:** Primer sequences for real-time PCR analysis

Gene	Primer sequences	Product size	GenBank accession no
*BMAL1*	F: 5′-CAGAAGCAAACTACAAGCCAA-3′	149 bp	NM_024362
	R: 5′-GGTCACATCCTACGACAAACA-3′		
*NR1D1*	F: 5′-CGGTCTACGGCAAGGCAACAC-3′	145 bp	NC_051345
	R: 5′-TTCTACCACCTCCCGCACAGC-3′		
*CRY1*	F: 5′-GTGGTGGCGGAAACTGCTCTC-3′	146 bp	NC_051342
	R: 5′-TGCGTCCTCTTCCTGACTTGGG-3′		
*PER3*	F: 5′-CAACCGCACCATCCGCAGAC-3′	82 bp	NM_023978
	R: 5′-CTTACGCCAGACGCCATGCTC-3′		
*GAPDH*	F: 5′-GATGCCCCCATGTTTGTGAT-3′	150 bp	NC_051339
	R: 5′-GGCATGGACTGTGGTCATGAG-3′		

### Statistics

Data results are all presented as mean ± s.e.m.. Differences between two groups were analyzed using the unpaired two-tailed Student’s *t* test or with Welch’s correction or using the Mann-Whitney test. Differences between more than two groups were analyzed by two-way ANOVA followed by a Tukey multiple-comparisons post hoc test or Kruskal-Wallis test with Dunn's correction. The two-way ANOVA was used with the following factors being considered: LD cycle (LD vs DL), model (normal vs OA), and their interaction LD × model. Values with *P* < 0.05 were considered to be statistically significant. Prism.7.00 (GraphPad Software Inc., USA) was used to perform all statistical analyses. SPSS 26 (IBM Ltd., USA) was used to analyze the significance of the slope of the linear regression equation of the body weight gain and the modeling week of rats in each group.

## Results

### DL cycle significantly inhibits the growth of adolescent rats

In this study, DL pattern significantly inhibited weight gain in normal and OA rats, and the rats subjected to ACLT showed more limited weight gain than normal+DL rats (Fig. 1B). By linear regression analysis on the week and the weight gain value of rats, there was a very significant difference in the slope of the fitted line between normal+LD and normal+DL groups, OA+LD and OA+DL groups, and the normal+DL and OA+DL groups (Fig. 1C). This indicates that DL cycle significantly slows down the body weight gain of rats. However, no significant difference in serum GH content was found among the four groups (Additional file 1). In addition, DL decreased the length of the total growth plate and the hypertrophy zone (HZ) in normal rats and OA rats (Fig. 1D), indicating that the DL cycle significantly inhibited the growth of rats independently of OA.

### Joint deterioration is markedly suppressed by the DL cycle

**Fig. 2:**
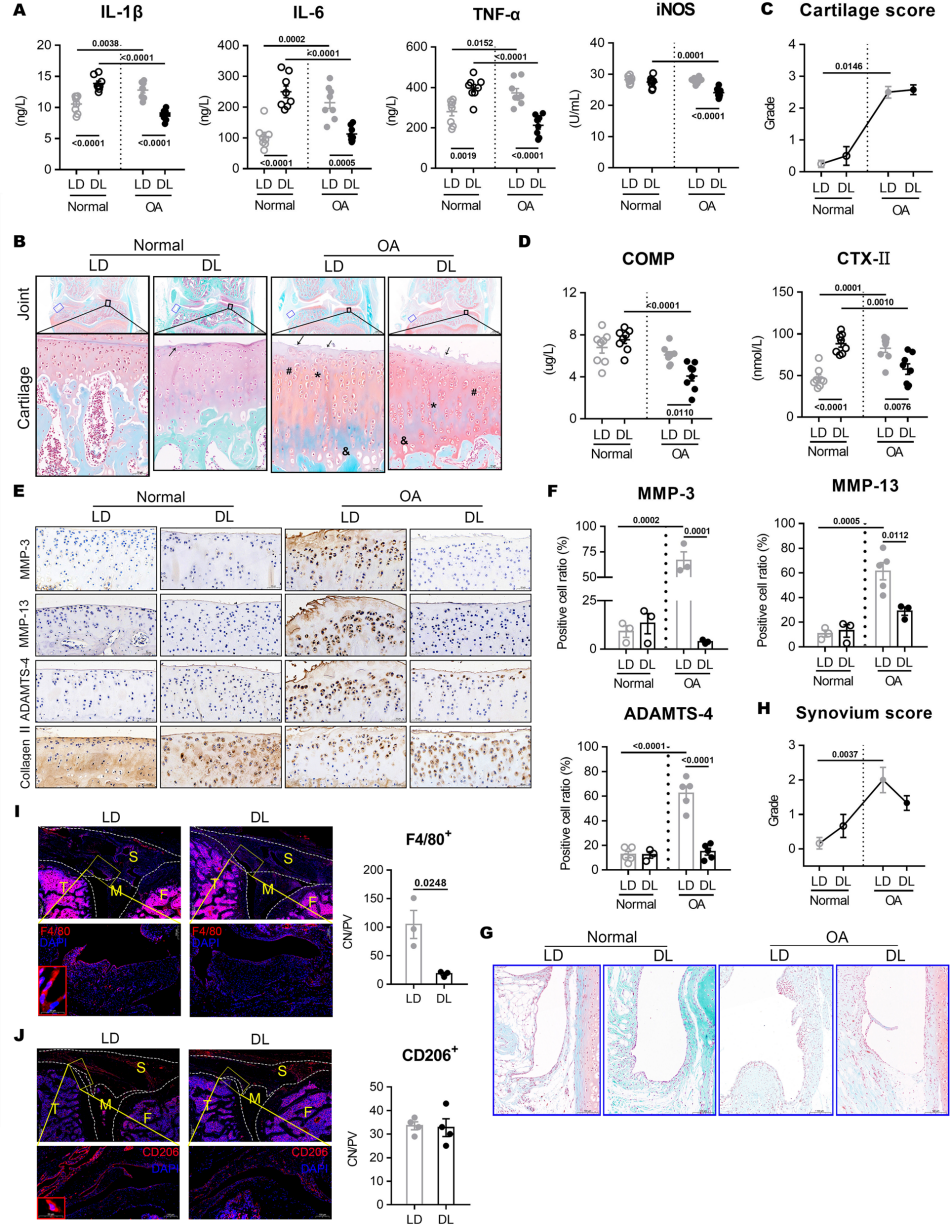
Effects of LD and DL cycle on cartilage and synovium pathology and inflammation in rats. **A** Concentration of pro-inflammatory factors of rat serum by ELISA. Two-way ANOVA: IL-1β: model factor: *F*_(1, 28)_ = 10.79, *P* = 0.0027; LD × model: *F*_(1, 28)_ = 75, *P* < 0.0001; IL-6: LD × model: *F*_(1, 28)_ = 62.04, *P* < 0.0001; TNF-α: model factor: *F*_(1, 28)_ = 5.127, *P* = 0.0315; LD × model: *F*_(1, 28)_ = 46.94, *P* < 0.0001; iNOS: LD factor: *F*_(1, 28)_ = 24.05, *P* < 0.0001; model factor: *F*_(1, 28)_ = 15.34, *P* = 0.0005; LD × model: *F*_(1, 28)_ = 10.89, *P* = 0.0026; *n* = 8 per group. **B**, **C** Safranin O and fast green staining of entire right knee joint and OARSI cartilage score. Scale bar, 1000 μm and 50 μm. Kruskal-Wallis test with Dunn’s correction: *n* = 3 to 6 per group. **D** Serum levels of COMP and CTX-II. Two-way ANOVA: COMP: model factor: *F*_(1, 28)_ = 24.27, *P* < 0.0001; LD × model: *F*_(1, 28)_ = 10.68, *P* = 0.0029; CTX-II: LD × model: *F*_(1, 28)_ = 43.8, *P* < 0.0001; *n* = 8 per group. **E**, **F** Immunohistochemical analysis in tibial cartilage. Scale bar, 50 μm. Two-way ANOVA: MMP-3: LD factor: *F*_(1, 8)_ = 33.23, *P* = 0.0004; group factor: *F*_(1, 8)_ = 22.55, *P* = 0.0014; LD × model: *F*_(1, 8)_ = 43.19, *P* = 0.0002; MMP-13: LD factor: *F*_(1, 10)_ = 6.197, *P* = 0.0320; group factor: *F*_(1, 10)_ = 29.78, *P* = 0.0003; LD × model: *F*_(1, 10)_ = 8.117, *P* = 0.0173; ADAMTS-4: LD factor: *F*_(1, 14)_ = 36.19, *P* < 0.0001; group factor: *F*_(1, 14)_ = 43.47, *P* < 0.0001; LD × model: *F*_(1, 14)_ = 35.41, *P* < 0.0001; *n* = 3 to 5 per group. **G**, **H** Safranin O and fast green staining of synovium. Scale bar, 100 μm. Kruskal-Wallis test with Dunn’s correction: *n* = 3 to 6 per group. **I**, **J** Immunofluorescence staining of F4/80^+^ and CD206^+^macrophages in synovium of OA rats. S, synovium; T, tibia; *F*, femur; M, meniscus. Scale bar, 100 μm and 500 μm. *n* = 3 per group

Inflammatory cytokines and active substances in peripheral blood circulation in each group as well as arthropathology were assessed to investigate whether DL cycle affects the inflammatory response and cartilage degeneration in normal and OA rats. ACLT induced a significant increase in IL-1β, IL-6, and TNF-α secretion under normal LD cycle condition (Fig. 2A). Surprisingly, DL significantly increased the secretion of these pro-inflammatory factors in normal rats, but it inhibited them in OA rats. The pathological changes of cartilage in OA rats (OA+LD) were more severe than those in the normal group (normal+LD) without altering the environment (Fig. 2B and C). An evident edema and the marked absence of cationic staining (asterisk) of the cartilage matrix and superficial fibrillation and abrasion (arrow) were found in OA rats. DL cycle caused a significant cartilage edema and surface discontinuity in normal rats (arrow), but it did not worsen the deterioration of cartilage or higher Osteoarthritis Research Society International (OARSI) score in OA rats (OA+DL). Consistently, the percentage of positive cells for the degradation enzymes of cartilaginous matrix, such as MMP-3, MMP-13, and ADAMTS-4, significantly increased after ACLT, while were dramatically reduced after DL cycle (Fig. 2D, E). Moreover, ACLT and DL cycle significantly reduced the collagen II content in cartilage. The DL cycle reduced the concentration of CTX-II and COMP in OA rat serum, which are metabolic markers of cartilage degradation [19] (Fig. 2F).

Additionally, DL cycle was not influencing the synovial pathology in OA rats but reduced partially the aggregation of inflammatory cells in the synovial layer (Fig. 2G and H). F4/80^+^ (pan-macrophage marker) macrophages markedly decreased in OA rat synovium under DL cycle (Fig. 2I), but no changes of CD206^+^ (M2 type) cells (Fig. 2J) were observed. These results suggested that the DL cycle significantly reduced the systemic and intra-articular inflammatory response in OA rats but activated the systemic inflammatory response in normal rats.

### DL cycle disturbs bone metabolism and accelerates bone resorption in OA rats

**Fig. 3:**
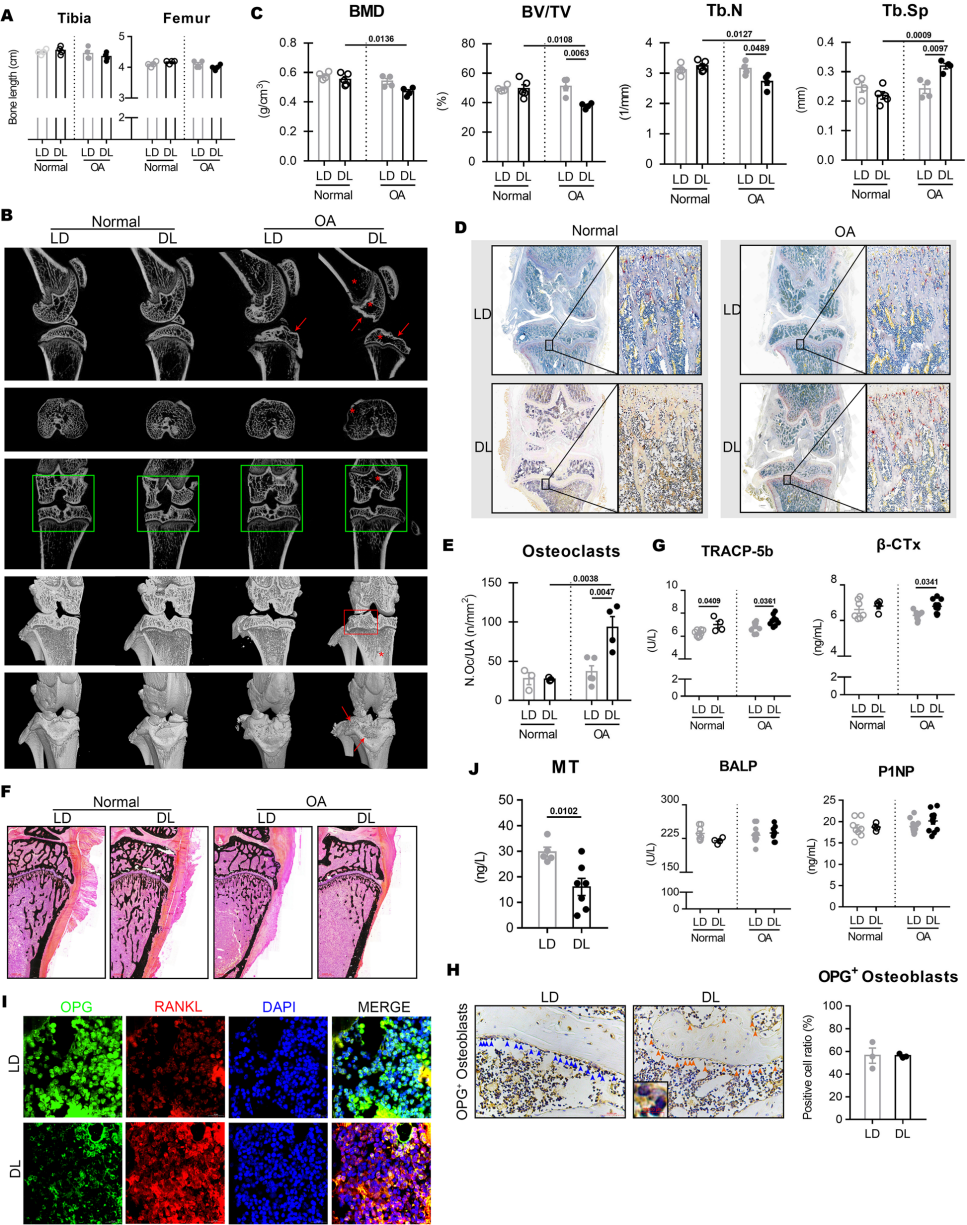
Effects of bone metabolism of rats under LD and DL cycle. **A** Femur and tibia lengths. *n* = 4 per group. **B**, **C** μCT scan and 3D images of femur and tibia epiphyses. Green boxed areas indicate the ROI selected for measurement and 3D microCT reconstruction. BV/TV, Tb.Th, Tb.N, Tb.Sp, and BMD were measured. Two-way ANOVA: BV/TV: LD factor: *F*_(1, 13)_ = 8.841, *P* = 0.0108; model factor: *F*_(1, 13)_ = 5.106, *P* = 0.0417; LD × model: *F*_(1, 13)_ = 8.672, *P* = 0.0114; Tb.N: model factor: *F*_(1, 13)_ = 5.445, *P* = 0.0363; LD × model: *F*_(1, 13)_ = 7.538, *P* = 0.0167; Tb.Sp: model factor: *F*_(1, 13)_ = 11.41, *P* = 0.0049; LD × model: *F*_(1, 13)_ = 14.32, *P* = 0.0023; BMD: LD factor: *F*_(1, 13)_ = 8.137, *P* = 0.0136; model factor: *F*_(1, 13)_ = 13.33, *P* = 0.0029; *n* = 4 to 5 per group. **D**, **E** TRAP staining and histomorphometric of rats. Scale bar, 1000 μm and 100 μm. Two-way ANOVA: LD factor: *F*_(1, 11)_ = 7.732, *P* = 0.0179; model factor: *F*_(1, 11)_ = 14.15, *P* = 0.0031; LD × model: *F*_(1, 11)_ = 8.118, *P* = 0.0158; *n* = 3 to 5 per group. **F** Von Kossa staining of undecalcified sections of tibias. Scale bar, 50 mm. **G** Concentration of TRACP-5b, β-CTx, P1NP and BALP in rat serum. Two-way ANOVA: TRACP-5b: LD factor: *F*_(1, 26)_ = 16.08, *P* = 0.0005; model factor: *F*_(1, 26)_ = 6.982, *P* = 0.0138; β-CTx: LD factor: *F*_(1, 26)_ = 5.751, *P* = 0.0240; *n* = 4 to 12 per group. **H** OPG^+^ osteoblasts in the epiphyseal trabecular bones of OA rats. Scale bar, 50 mm. *n* = 3 per group. **I** Immunofluorescence staining of bone marrow stromal cells in OA rats. Green, OPG^+^ cells; red, RANKL^+^ cells; blue, nucleus. Scale bar, 20 μm. **J** Levels of MT in OA rat serum. *n* = 5-7

The lengths of both the femur and tibia of rats showed no statistical difference among four groups (Fig. 3A, Additional file 2). Femoral and tibial subchondral bone in OA rats showed evident surface unevenness by μCT scan of the hindlimbs (Fig. 3B, arrow) compared with normal rats. Furthermore, a significant decrease in the trabecular bone was found in OA rats (asterisk) with thinner articular bone plate and epiphyseal plate (frame) under DL cycle by micro-CT and 3-dimensional (3D) reconstruction. Similarly, an evident decrease in bone mineral density (BMD), percent bone volume with respect to total bone volume (BV/TV) and trabecular number (Tb.N), as well as an increase in trabecular separation (Tb.Sp) in OA+DL rats were found (Fig. 3C). TRAP staining showed that the number of osteoclasts in normal rat bone tissue under DL cycle was not different from that under LD cycle, but dramatically increased in OA rats (Fig. 3D, E). In addition, the Von Kossa staining area was significant reduced in OA rats under DL cycle, indicating a decrease in the density of calcification of the trabeculae bone of the hind limbs (Fig. 3F).

Several classical serum markers were measured to further evaluate the DL pattern on bone metabolism (Fig. 3G). The content of some bone resorptive markers TRACP-5b and β-CTx was increased under DL cycle than under LD cycle in OA rats, but not different from normal rats. However, the levels of the bone formation markers P1NP and BALP did not significantly differ among each group and neither did OPG^+^ osteoblasts (Fig. 3H, blue arrow). This result indicated that the capacity of bone resorption was enhanced under DL condition, but not the bone formation. Moreover, OPG secretion was clearly suppressed in bone stromal cells/osteogenic precursor cells of the OA+DL rats (Fig. 3I). RANKL expression was abnormally increased under DL cycle in OA rats, which enhanced the osteoclastogenesis. Furthermore, the DL cycle significantly inhibited the secretion of MT (Fig. 3J). These results fully suggested that the DL cycle significantly disturbed the balance of bone metabolism, and made the state biased towards the development of bone resorption in OA rats.

### DL cycle activates the myeloid differentiation of the mononuclear/phagocyte system in OA rat skeletal system

**Fig. 4:**
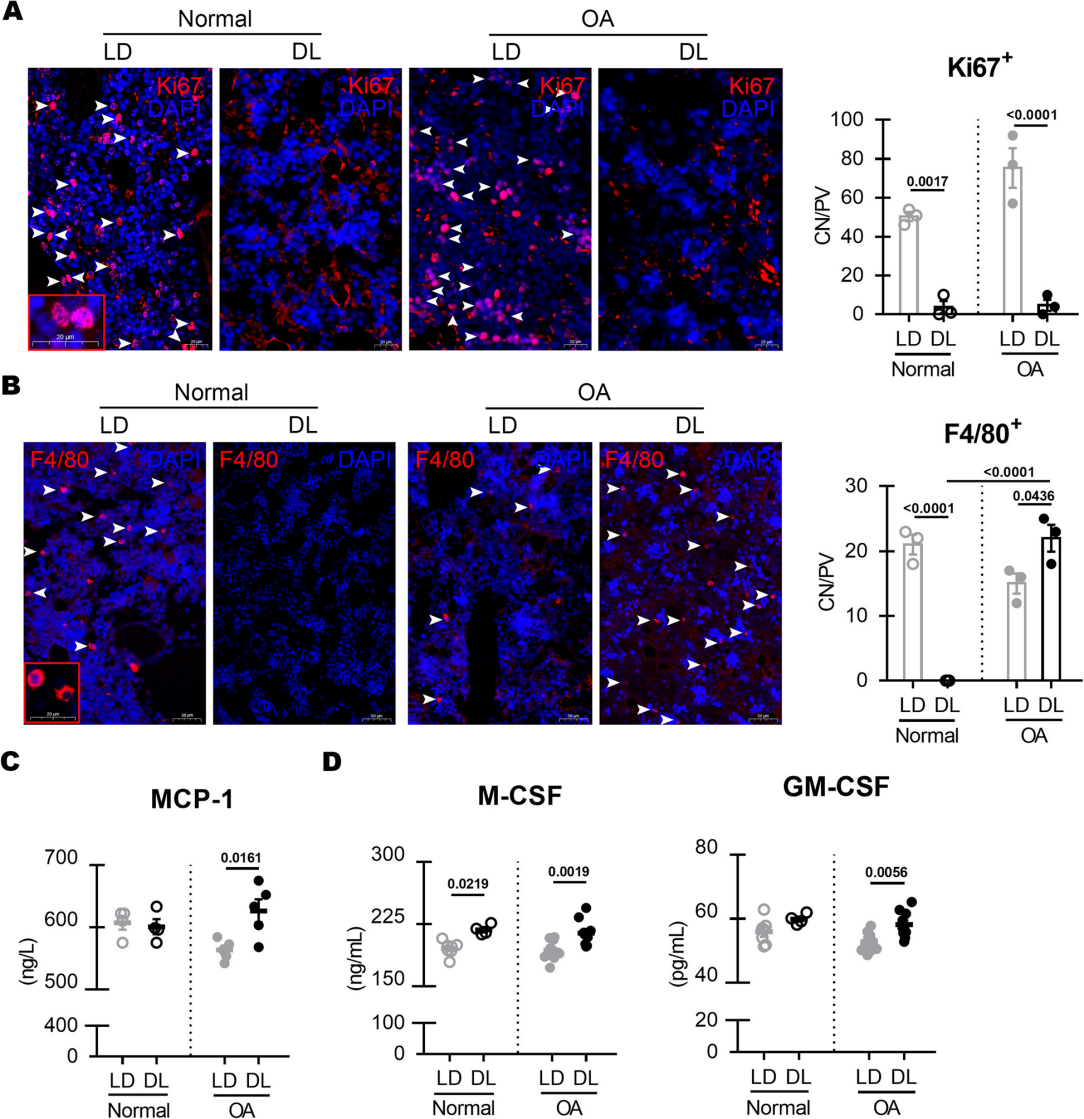
Significant activity of the bone marrow MPS after LD reversal. **A** Presentative images of proliferation of tibial marrow cells stained by Ki67 (white arrow) and DAPI. Scale bar, 50 μm and 20 μm. Two-way ANOVA: LD factor: *F*_(1, 8)_ = 108.6, *P* < 0.0001; model factor: *F*_(1, 8)_ = 5.332, *P* = 0.0498; *n* = 3 per group. **B** Presentative images of F4/80^+^ macrophages (white arrow) of tibia. Scale bar, 50 μm. Two-way ANOVA: LD factor: *F*_(1, 8)_ = 21.78, *P* = 0.0016; model factor: *F*_(1, 8)_ = 28.44, *P* = 0.0007; LD × model: *F*_(1, 8)_ = 87.11, *P* < 0.0001; *n* = 3 per group. **C**, **D** Concentration of MCP-1, GM-CSF and M-CSF in rat serum. Two-way ANOVA: MCP-1:LD × model: *F*_(1, 14)_ = 6.652, *P* = 0.0218; M-CSF: LD factor: *F*_(1, 28)_ = 23.57, *P* < 0.0001; GM-CSF: LD factor: *F*_(1, 28)_ = 12.88, *P* = 0.0013; *n* = 4 to 11 per group

Since the DL cycle abnormally increased osteoclasts in OA rats, our hypothesis was that this condition could affect cell differentiation in the skeletal system. The bone marrow cells under proliferation were assessed by the staining of the Ki67 protein. The DL cycle significantly inhibited marrow cell proliferation in both normal and OA rat bones (Fig. 4A). The DL cycle also significantly reduced F4/80^+^ macrophages in normal skeleton consistent with the trend of macrophages in synovium and Ki67^+^ cells in bone marrow (Fig. 4B, C). On the contrary, DL markedly stimulated the production of F4/80^+^ and CD206^+^ macrophages in OA rats (Fig. 4B, C; Additional file 3). The concentration of MCP-1, M-CSF, and GM-CSF in the circulatory system of OA rats under DL cycle was significantly higher than that of in the OA+LD rats (Fig. 4D, E). These results indicated that mononuclear/phagocyte system (MPS) in the bone marrow of OA rats was significantly activated by the DL condition.

### LD inversion disturbs the expression of circadian clock proteins in body OA rats

**Fig. 5:**
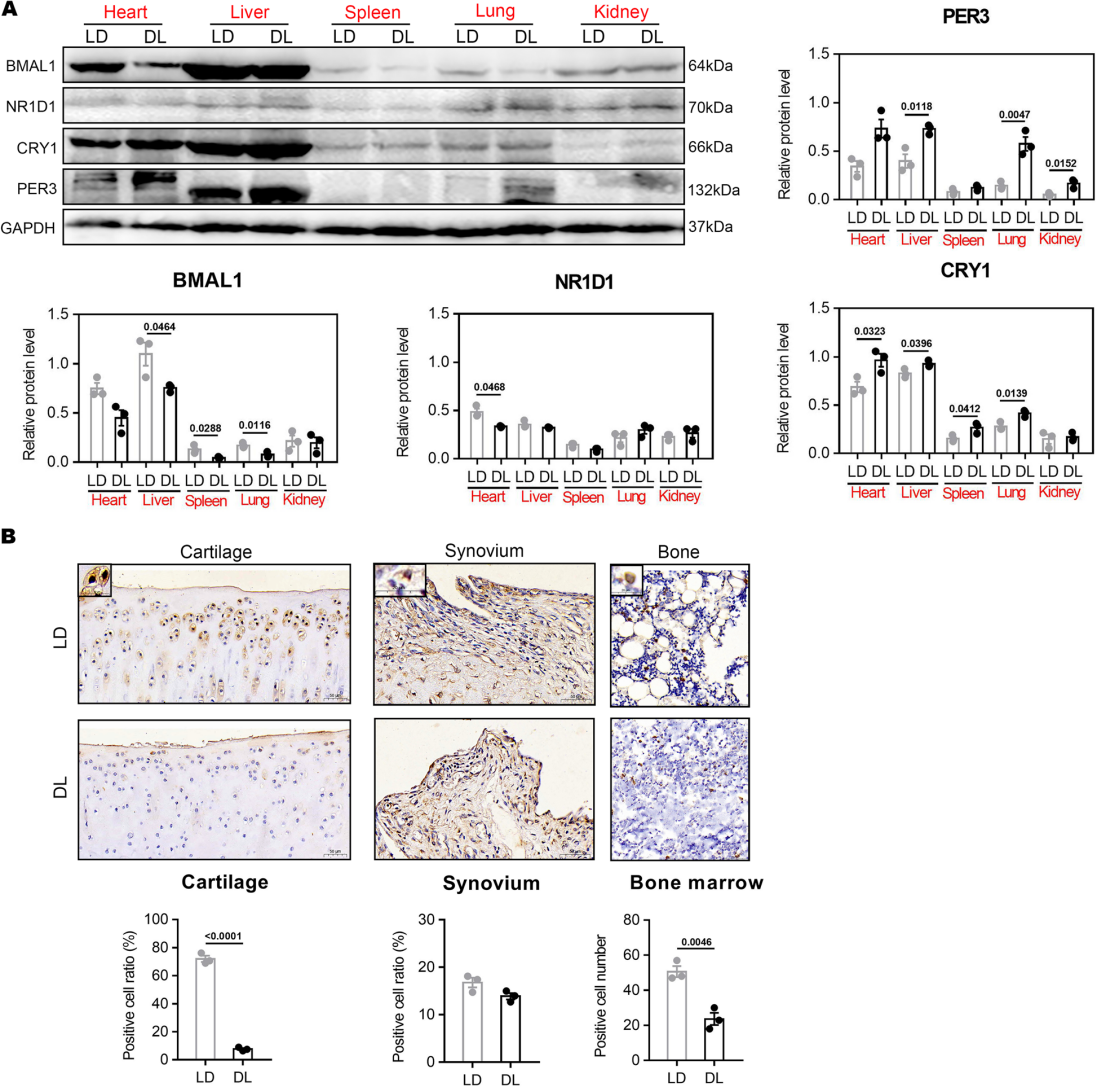
Changes in expression of clock proteins in representative tissues. **A** Expression of BMAL1, NR1D1, CRY1, and PER3 protein in heart, liver, spleen, lung, and kidney of OA rats via WB. Unpaired two-tailed Student’s *t* test or with Welch’s correction, or using the Mann-Whitney test: *n* = 3 per group. **B** Expression of BMAL1 protein of tibial cartilage, synovium, and bone marrow of OA rats via IHC staining. Scale bar, 50 μm. *n* = 3 per group

Several previous studies showed that shifted LD cycles or BMAL1 knockout disrupts the circadian system of mice, which showed low bone mass phenotype [20–22]. In this study, BMAL1 protein was significantly reduced in heart, liver, spleen, and lung tissues of OA+DL rats compared with that in OA+LD rats (Fig. 5A), while CRY1 and PRE3 were markedly increased. The mRNA level of BMAL1, CRY1, and PER3 in these tissues also showed a corresponding alteration (Additional file 4). Additionally, the expression of BMAL1 protein in the hind limbs was significantly decreased in bone marrow and cartilage under the LD cycle, but not in the synovium (Fig. 5B). These results indicated the disturbance of the biological clock in OA rats under DL cycle.

## Discussion

This study demonstrated that continuous DL cycle affected bone turnover and structure. However, changes in cortical bone volume and thickness were not detected. It is well known that irregular LD cycles or circadian rhythm disorders (CRD) cause bone loss [23]. The results of this study are consistent with the effects of LL exposure on the skeletal system. LL 24 h for 24 weeks induces the bone trabecular volume and number in mice, which is manifested as early osteoporosis [24]. A smaller tibia tarsus cortical area and mean cortical thickness are found in chicken under sustained LL [25]. However, different patterns of light or dark radically affect bone health. DD 24 h predates tibia and femur ossification in chicken embryos [25]. Schilperoort et al. established 10 weeks with a weekly alteration of the light condition (12 h shifts; LD-DL) in mice and found that bone formation and resorptive capacity are significantly reduced, but the number of osteoblasts and osteoclasts is not different [26]. Our study clearly demonstrated a different conclusion, probably because of differences in the LD cycle pattern, or because the animal was inflamed. Some typical clock gene deletions result in remarkable effects on the skeletal system [22, 27]. Moreover, the number of active osteocytes and osteoblasts in *BMAL1*^*-/-*^ mice was significantly reduced [28, 29]. The RANKL/RANK/OPG system plays an important role in regulating osteoclastogenesis and osteoblast function; the expression of RANK and RANKL markedly increases in the bone of *BMAL1*^*-/-*^ mice, along with the increase of osteoclasts parameters [22]. In this study, DL significantly increased the ratio of RANKL/OPG, but no significant effect on the number of osteoblasts and bone formation function was found. These results further confirmed that DL induced bone resorption rather than bone formation.

The body’s rhythm runs freely under constant conditions, while the disrupted LD pattern or jet lag induces CRD [20]. This raises the question of whether DL cycle causes abnormal bone metabolism through an endogenous biorhythm disorder. However, one study has showed that continuous LL weakens the behavior and SCN rhythm of mice [24]. Dudek et al. proved no significant difference in rhythmic activity patterns between the wildtype (WT) and *BMAL1*-cKO mice when exposed to 12 h/12 h LD or DD [30]. Therefore, further studies are needed to confirm whether OA rats are entrained or better adapted to DL conditions. The central nervous system regulates the homeostasis of the body's internal environment by continuously releasing various hormones into the peripheral blood circulation at a relatively balanced speed and rhythm, coordinating with the endocrine system. Circadian rhythms also regulate bone metabolism through the sympathetic nervous system and a variety of hormones, such as the glucocorticoid hormone, MT, and parathyroid hormone [31, 32]. MT enhances osteogenesis, suppresses osteoclast formation and activation [33, 34], and participates in bone turnover. This study found that DL significantly reduced the level of MT in OA rats, which was theoretically consistent with the dramatic increase in the number and function of osteoclasts.

MPS is a major component involved in specific immune responses that secretes a variety of bioactive substances (lysozyme, complement, IL-1, TNF-α) to participate in inflammatory responses. In this study, the inflammatory response of OA rats was significantly reduced under continuous DL. However, inflammation was intense in normal rats under DL cycle. Bunger et al. [35] found that *Mop3*^*-/-*^ mice present typical non-inflammatory arthropathy after more than 20 weeks but with progressive calcification of tendons and ligaments and heterotopic ossification, whereas most studies showed that chronic shifted LD cycle increases inflammation and pathological changes in normal cartilage [11, 36]. An impaired molecular clock, sleep deprivation, and shifting light-dark patterns affect the circulating white blood cells, causing leukocytosis and an increase in inflammatory cytokine levels [37]. Therefore, why does DL trigger the opposite effect in the presence of chronic inflammation in the body? Why the MPS system is still significantly activated after the significant suppression of the inflammatory response in OA rats? One hypothesis is that this may be related to the polarization of macrophages. Macrophages are polarized to different phenotypes under the stimulation of different cytokines and chemokines [38]. Additionally, Aiello et al. demonstrated that chronic jet lag reduces the M1/M2 ratio in the tumor microenvironment, facilitating tumor growth [39]. According to our results, DL altered the joint and bone marrow microenvironmental homeostasis in OA rats, showing polarization toward the M2 phenotype (non-inflammatory) rather than M1, thus inducing different immune responses. Our further studies might improve and supplement these results.

The limitations of this study include that the locomotor assay and circadian expression rhythm amplitude of the intrinsic molecular clock were not performed in experimental rats due to limited experimental conditions and sample size. Therefore, we failed to demonstrate the circadian rhythm changes of OA rats under DL condition. Besides, chondropathology and cartilage-bone crosstalk, such as changes of vascularization and innervation of calcified cartilage, may play an important role in distinct changes in bone structure.

Some studies confirmed that the biological clock and arthritis interact with each other, and the interplay influences human health and diseases [40, 41]. A pharmacological therapy targeting clock proteins showed a significant effect in alleviating OA pain [42]. Light therapy is used to improve sepsis and organ damage in mice [43, 44]. It has become common for people, especially teenagers, to suffer from sleep disturbances or social jetlag due to the accelerated pace of life and work. Timely understanding of the risk factors is necessary to prevent or manage OA. It is of utmost importance to prevent the occurrence of osteoporosis induced by CRD in postmenopausal women. More recently, Winter et al. suggested that strategies focusing on preventing osteoporosis should include lifestyle interventions rather than expensive and often side-effect medication [45]. Overall, a considerable value should be placed on the negative effects of the light-dark cycle on our lives and health.

## Conclusions

Dark-light cycle disturbs bone metabolism and inhibits arthritis in osteoarthritic rats. Clock proteins in osteoarthritic rats are is also abnormally expressed.

## Supplementary Information

**Additional file 1. **Level of GH in normal and OA rat serum of each group by ELISA. Repeated measures two-way ANOVA; *n*=3 per group.

**Additional file 2.** Photography of rat femurs and tibias in each group.

**Additional file 3. **Number of CD206^+^ bone marrow cells of OA rats in LD and DL condition. Immunofluorescence staining for CD206^+^ cells of OA rats. Number of positive cells per field of view was counted and analyzed by unpaired Student’s t test. Scale bar, 1000 μm and 50 μm (magnified images) *n* = 4 per group.

**Additional file 4. **Changes in levels of clock genes in representative tissues. Relative mRNA level of BMAL1, NR1D1, CRY1, and PER3 in heart, liver, spleen, lung, and kidney in OA rats under LD and DL cycle via qPCR. Unpaired Student’s t test or Mann-Whitney test were used, or with Welch’s test for correction; *n* = 3-4 per group.

## Data Availability

The datasets used and/or analyzed during the current study are available from the corresponding author on reasonable request. All data generated or analyzed during this study are included in this published article [and its supplementary information files].

## Abbreviations

ACLT: Anterior cruciate ligament transection
ADAMTS: A disintegrin and metalloproteinase with thrombospondin motifs
BALP: Bone alkaline phosphatase
BMAL1: Brain and muscle Arnt-like protein 1
BMD: Bone mineral density
CMP: Common myeloid progenitors
COMP: Cartilage oligomeric matrix protein
CRD: Circadian rhythm disorders
CRY1: Cryptochrome 1
CTX-II: C-Terminal telopeptides of collagen type II
GAPDH: Glyceraldehyde-3-phosphate dehydrogenase
GH: Growth hormone
GM-CSF: Granulocyte-macrophage colony stimulating factor
HZ: Hypertrophy zone
IL-1β: Interleukin-1β
iNOS: Inducible nitric oxide synthase
LD: Light and dark
MCP-1: Monocyte chemoattractant protein-1
MMP: Matrix metalloproteinase
MT: Melatonin
NR1D1: Nuclear receptor subfamily 1 group D member 1
OA: Osteoarthritis
OPG: Osteoprotegerin
PER3: Period 3
PTH: Parathyroid hormone
P1NP: N-Terminal propeptide of type 1 collagen
RA: Rheumatoid arthritis
RANKL: Receptor activator of nuclear factor-kappa B ligand
RHT: Retina hypothalamic tract
SCN: Suprachiasmatic nucleus
Tb.N: Trabecular number
Tb.Sp: Trabecular separation
TRAP: Tartrate-resistant acid phosphatase
TNF-α: Tumor necrosis factor α
β-CTx: β-Isomerized C-terminal telopeptides
