# Supplementary figures and images for: Dark-light cycle disrupts bone metabolism and suppresses joint deterioration in osteoarthritic rats
Source: Arthritis Res Ther. 2022 Jun 28;24:158. doi: 10.1186/s13075-022-02832-8 (PMC9238010; doi:10.1186/s13075-022-02832-8)

**
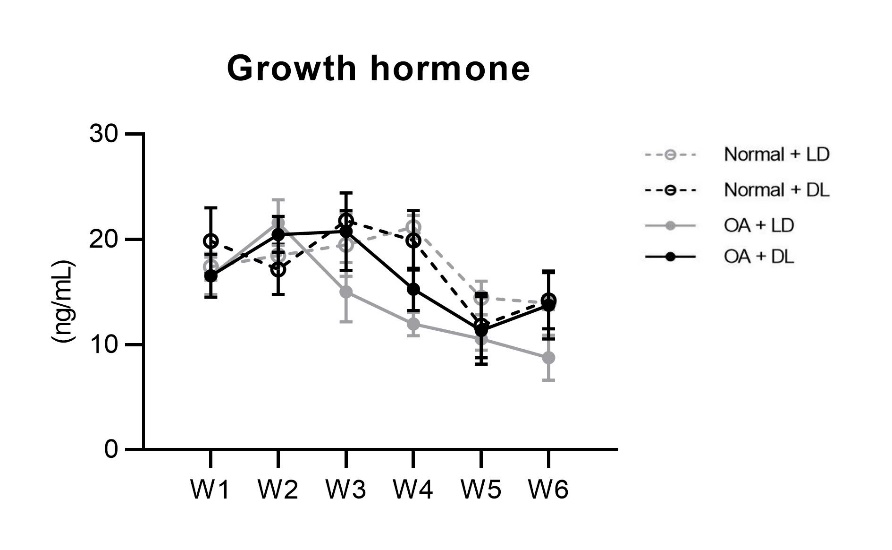
**

Supplement: Supplementary file 1 — Additional file 1. Level of GH in normal and OA rat serum of each group by ELISA. Repeated measures two-way ANOVA; n=3 per group. [file 13075_2022_2832_MOESM1_ESM.docx]

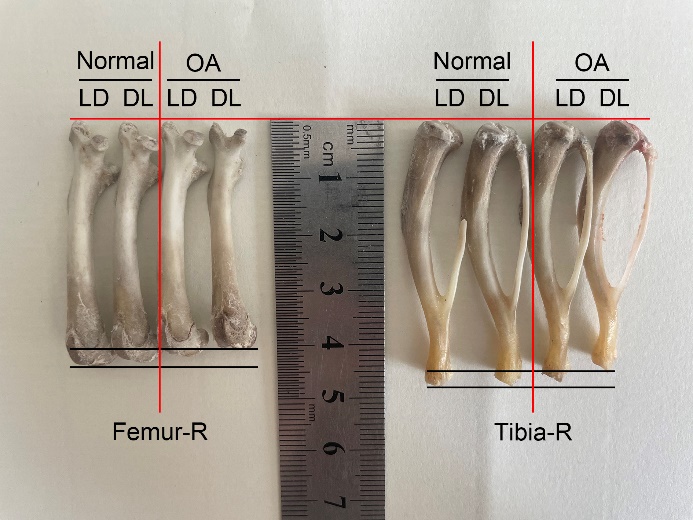

Supplement: Supplementary file 2 — Additional file 2. Photography of rat femurs and tibias in each group. [file 13075_2022_2832_MOESM2_ESM.docx]

**
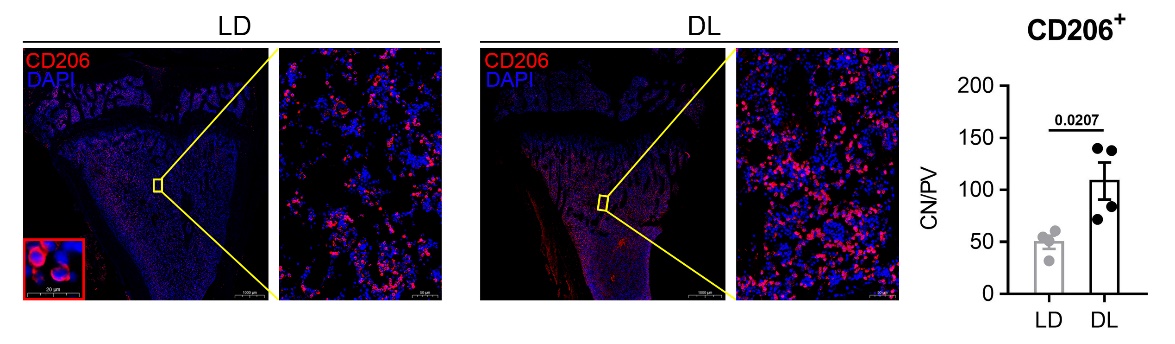
**

Supplement: Supplementary file 3 — Additional file 3. Number of CD206+ bone marrow cells of OA rats in LD and DL condition. Immunofluorescence staining for CD206+ cells of OA rats. Number of positive cells per field of view was counted and analyzed by unpaired Student’s t test. Scale bar, 1000 μm and 50 μm (magnified images) n = 4 per group. [file 13075_2022_2832_MOESM3_ESM.docx]

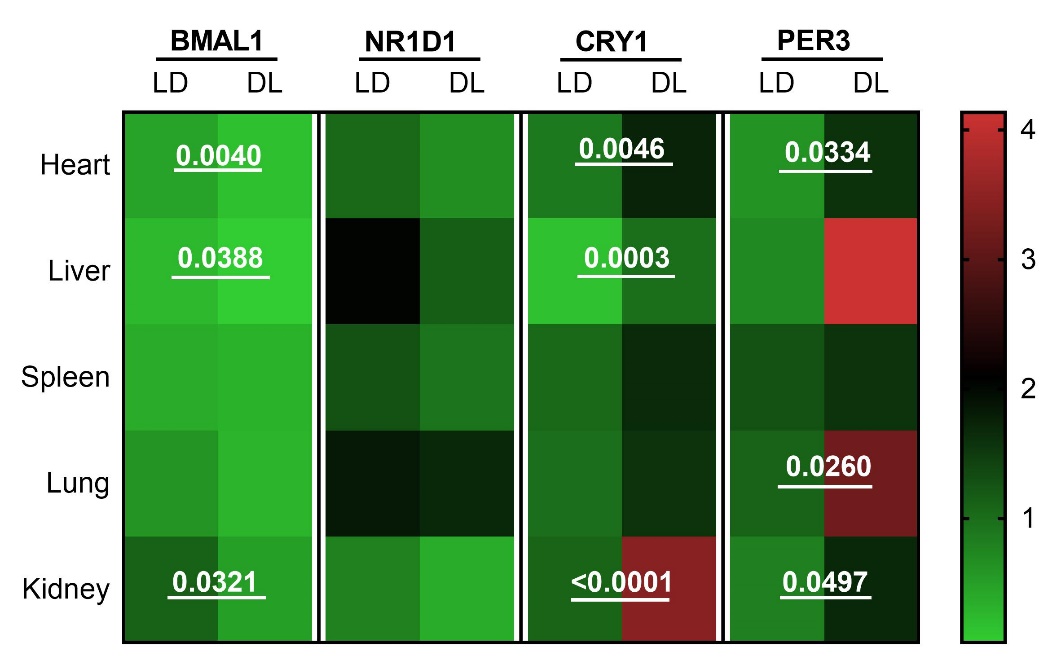

Supplement: Supplementary file 4 — Additional file 4. Changes in levels of clock genes in representative tissues. Relative mRNA level of BMAL1, NR1D1, CRY1, and PER3 in heart, liver, spleen, lung, and kidney in OA rats under LD and DL cycle via qPCR. Unpaired Student’s t test or Mann-Whitney test were used, or with Welch’s test for correction; n = 3-4 per group. [file 13075_2022_2832_MOESM4_ESM.docx]
